# Life-Saving Endovascular Management of a Ruptured Iatrogenic Infrarenal Aortic Pseudoaneurysm

**DOI:** 10.7759/cureus.82721

**Published:** 2025-04-21

**Authors:** Miguel A Peraza-Arjona, Victor M Ayuso-Diaz, Alfonso Peraza-Fernandez, Maria Elena Ayuso-Diaz, Angelica Moreno-Enriquez, José D Vargas-Gómez

**Affiliations:** 1 Angiology and Vascular and Endovascular Surgery, Elvia Carrillo Puerto Regional Hospital, Instituto De Seguridad Y Servicios Sociales de Los Trabajadores Del Estado, Yucatan, MEX; 2 Research and Education, Medical Care and Research, Yucatan, MEX; 3 Surgery, Elvia Carrillo Puerto Regional Hospital, Yucatan, MEX; 4 Genomic-Metabolic Unit, Marista University of Mérida, Yucatan, MEX; 5 Angiology and Vascular and Endovascular Surgery, Elvia Carrillo Puerto Regional Hospital, Yucatan, MEX; 6 Genomic-Metabolic Unit, Marista University of Merida, Yucatan, MEX; 7 Surgery, Agustin O'Horan General Hospital, Yucatan, MEX

**Keywords:** abdominal aorta, aortic injury, case report, emergency management, endovascular repair, iatrogenic injury, infrarenal pseudoaneurysm, ruptured pseudoaneurysm, vascular surgery, vascular trauma

## Abstract

Traumatic injuries to the aorta are rare but carry a high risk of mortality. In particular, penetrating injuries to the abdominal aorta pose a significant challenge as many patients do not survive long enough to reach the hospital. Although vascular complications during laparoscopic surgery are rare, they can occur during pneumoperitoneum or trocar insertion. Endovascular stenting is an established treatment for blunt aortic injuries, but its use in the management of penetrating injuries is less well-defined. We present the case of a 68-year-old woman who developed hypovolemic shock during an attempted laparoscopic cholecystectomy. The procedure was converted to open due to hemodynamic instability. Intraoperative findings revealed a retroperitoneal hematoma, and no active source of bleeding could be identified. Given the transient stabilization of the patient and the absence of visible ongoing bleeding, a conservative approach was adopted. Several months later, the patient experienced an episode of syncope, which prompted further imaging and led to the diagnosis of a ruptured iatrogenic abdominal aortic pseudoaneurysm. The patient was treated with endovascular deployment of a BeGraft endoprosthesis, and hemodynamic stability was successfully restored.

## Introduction

Iatrogenic pseudoaneurysm of the infrarenal abdominal aorta is a rare but potentially life-threatening complication, usually resulting from complex medical or surgical procedures. It occurs when a lesion in the arterial wall allows blood extravasation and the formation of a cavity in communication with the lumen without involving all layers of the vessel, which distinguishes it from a true aneurysm. Although the incidence is low, the incidence of iatrogenic pseudoaneurysms following percutaneous arterial punctures has been reported to be as high as 7%. In a recent study by Norwood et al., half of the pseudoaneurysms occurred after arterial catheterization, and one-third of the pseudoaneurysms were related to a previous surgical anastomosis; the clinical impact is significant, especially in the context of minimally invasive and high-risk procedures where adequate closure of the puncture site is essential to avoid complications [1-4].

In terms of pathogenesis, the formation of iatrogenic pseudoaneurysms is associated with an inflammatory process and deficiencies in tissue repair following vascular injury. Factors such as improper handling during the procedure, the use of inefficient closure devices, or excessive pressure on the endotracheal cuff during ventilation in the intensive care unit (ICU) may contribute to their development. Recent studies have shown that the pathological processes underlying pseudoaneurysm formation after endovascular interventions, although sharing common mechanisms of extracellular matrix degradation and inflammatory responses, have specific characteristics that require a unique diagnostic and therapeutic approach [3].

The diagnosis of these pseudoaneurysms requires a multimodality approach using advanced imaging modalities, such as computed tomography with angiography and vascular ultrasound, to accurately define the extent and morphology of the lesion. These modalities allow for the identification of discontinuities in the aortic wall, retroperitoneal hematomas, or signs of contrast extravasation, which are essential for therapeutic planning. In addition, the use of bedside ultrasound has been established as a useful tool in the emergency setting for the initial detection of aortic abnormalities, although it has limitations in the detection of ruptures [4,5].

## Case presentation

A 68-year-old woman with no history of chronic degenerative diseases was scheduled for laparoscopic cholecystectomy for cholelithiasis. During trocar insertion, she developed significant arterial bleeding, which resulted in hypovolemic shock (systolic blood pressure <90 mmHg, tachycardia >120 bpm, and cold extremities). Intraoperative packing was performed to control the bleeding. After 48 hours, hemodynamic instability persisted and reoperation was decided: an extensive retroperitoneal hematoma (approx. 15 × 10 cm) was found, with no evidence of active bleeding, and conservative management with close monitoring in the ICU was chosen. 

Over the next six months, the patient presented with progressive episodes of pallor, exertional dyspnea, headache and tachycardia, and recurrent orthostatic hypotension. A slow but steady decline in hemoglobin was observed (from 10 g/dL to 7 g/dL in ambulatory controls). Treatment with iron and folic acid was attempted on suspicion of occult gastrointestinal bleeding, although no lesions were found on endoscopy. Finally, the patient suffered a syncopal episode at home and was taken to the emergency department (ED) where he was found to have a hemoglobin of 4 g/dL, marked tachycardia (150 bpm), and severe hypotension (SBP < 80 mmHg).

Referral to angiology and vascular surgery was requested due to the hemodynamic instability and history of retroperitoneal hematoma. In addition to cutaneous and mucosal pallor, mild pain was noted in the left flank with no apparent pulsatile abdominal mass. Laboratory tests confirmed severe anemia and other relevant findings (Table 1). 

**Table 1:** Laboratory parameters at the time of admission to the emergency department (ED).

Parameter	Value	Reference range
Hemoglobin	4.0 g/dL	12.0-16.0 g/dL
Hematocrit	13%	36-46%
White blood cells (WBC)	14,500/mm³	4,000-11,000/mm³
C-reactive protein (CRP)	15 mg/dL	<1 mg/dL
Platelets	210,000/mm³	150,000-400,000/mm³
Blood group	O Positive	-
Total bilirubin	0.9 mg/dL	0.3-1.2 mg/dL
Direct bilirubin	0.2 mg/dL	0.0-0.3 mg/dL
Alkaline phosphatase (ALP)	70 U/L	30-120 U/L
Gamma-glutamyl transferase (GGT)	30 U/L	5-50 U/L
AST (glutamate oxaloacetate transaminase)	25 U/L	10-40 U/L
ALT (glutamate pyruvate transaminase)	30 U/L	7-56 U/L

**Figure 1:**
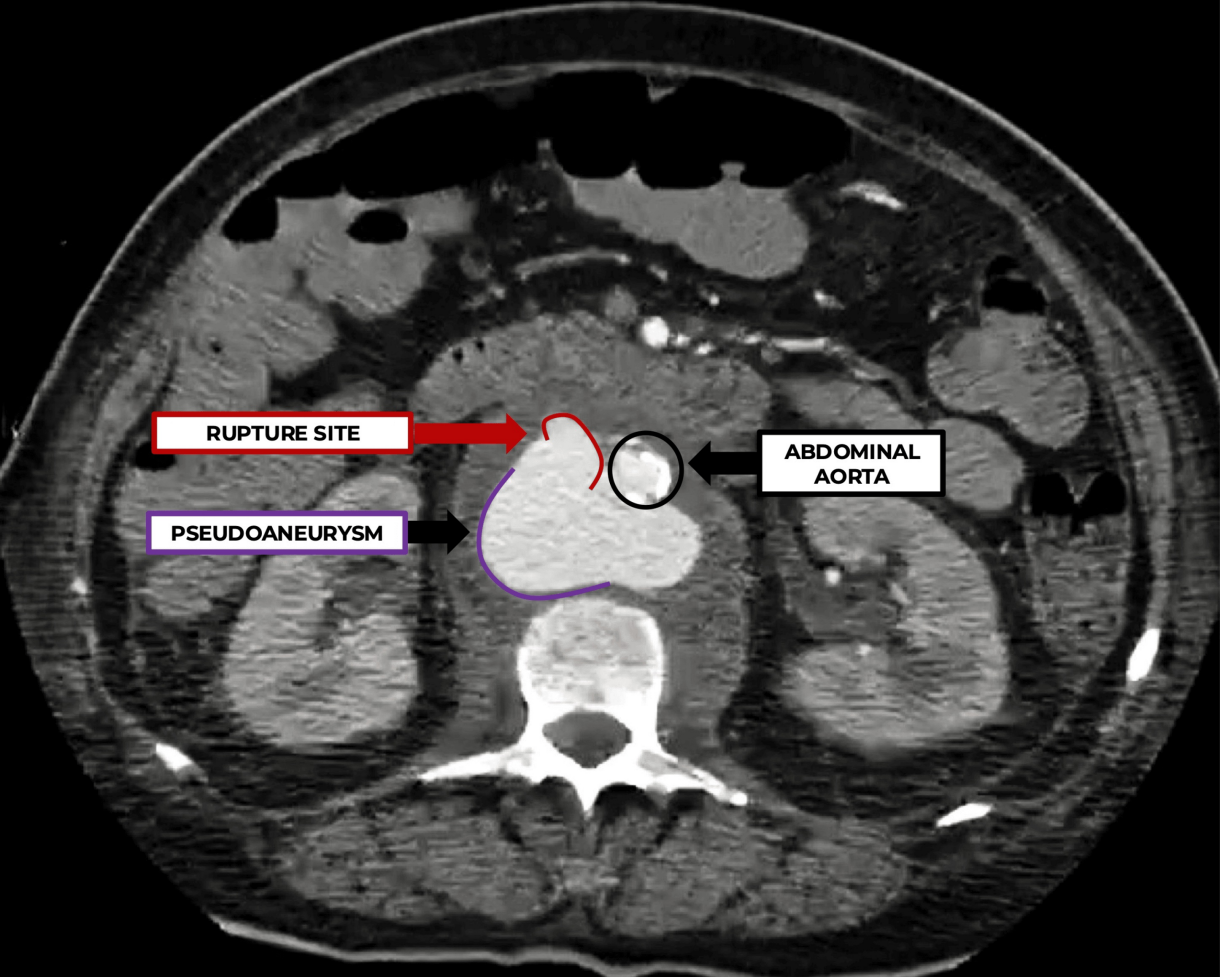
Abdominal angiotomography, axial view. Infrarenal pseudoaneurysm of the abdominal aorta.

Emergency abdominal angiotomography was performed due to high suspicion of a vascular complication. The axial section (Figure 1) showed an infrarenal abdominal aortic pseudoaneurysm measuring approximately 4.5 × 3.2 cm, partially contained by the retroperitoneal hematoma. 

**Figure 2:**
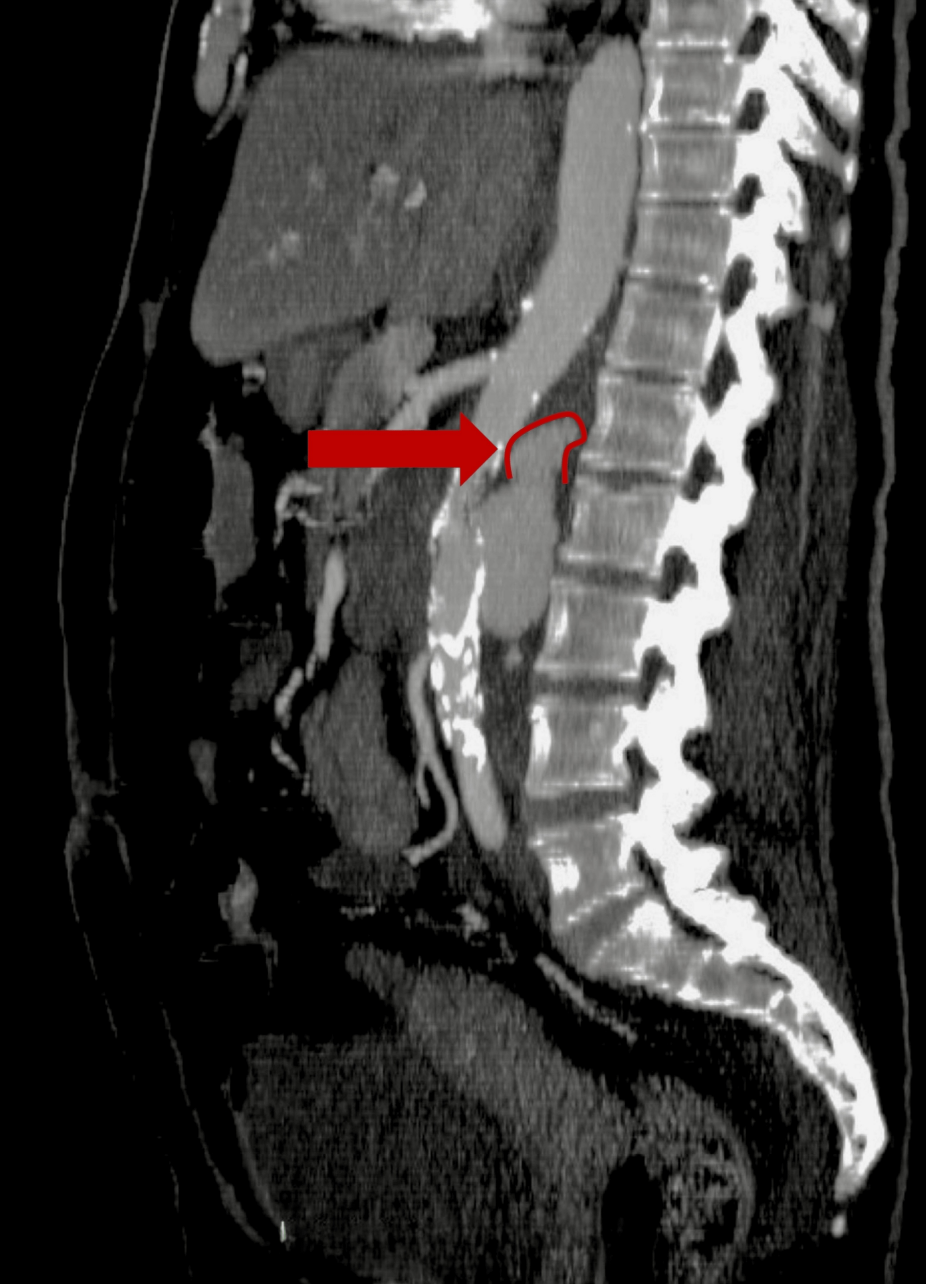
Abdominal angiotomy, sagittal section. The red arrow shows the rupture in the upper part of the pseudoaneurysmal sac.

Sagittal reconstruction (Figure 2) showed evidence of rupture in the upper part of the pseudoaneurysmal sac with extravasation of contrast into the retroperitoneal space. These findings confirmed the presence of a ruptured pseudoaneurysm of the abdominal aorta, probably related to the inadvertent injury during trocar placement six months earlier. 

**Figure 3:**
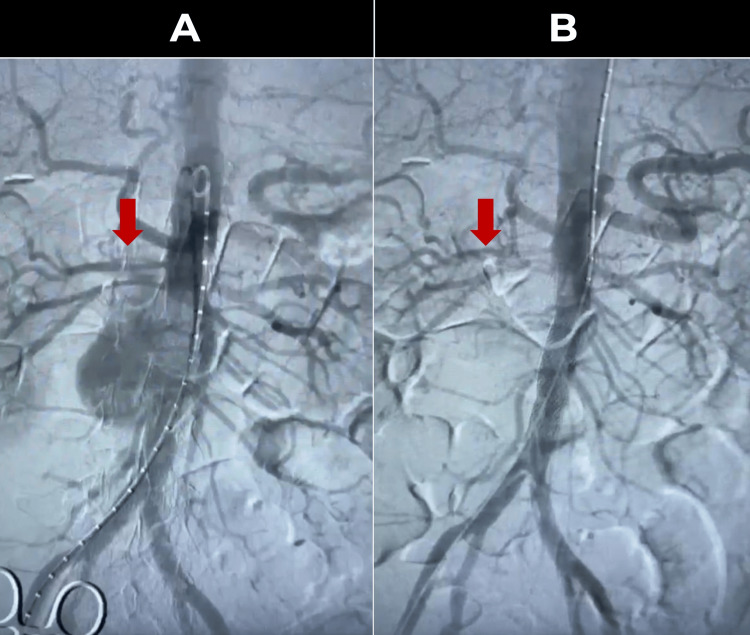
Comparative aortographies. A. Initial: Contrast extravasation is observed in the upper portion of the pseudoaneurysm (red arrow). B. Final: Proper placement of the stent is demonstrated, with no evidence of leakage and adequate contrast flow into both iliac arteries (red arrow).

Given the clinical deterioration and the infrarenal location of the pseudoaneurysm, an urgent endovascular approach was chosen. Under balanced general anesthesia and invasive monitoring, the right common femoral artery was punctured, and a long introducer advanced into the infrarenal aorta. A 16 × 58 mm BeGraft stent was implanted (Figure 3A), covering the ruptured area and sealing the leak. Control angiography (Figure 3B) confirmed the successful exclusion of the pseudoaneurysm, with adequate flow to the iliac arteries and no endoleaks. 

Throughout the procedure and for the first 24 hours after, the patient required a total of 10 red blood cell concentrates (six preoperative and four postoperative) to maintain hemoglobin above 8 g/dL. In addition, crystalloids and blood products were administered according to hemodynamic parameters and continuous monitoring. Coagulation studies showed a prothrombin time (PT) of 13.2 seconds, an international normalized ratio (INR) of 1.18, and an activated partial thromboplastin time (aPTT) of 31.5 seconds in the early postoperative period, indicating preserved coagulation function. Blood pressure gradually stabilized, vasopressin was discontinued after 12 hours, and inotropic support was withdrawn in less than 24 hours. The outcome was favorable, allowing discharge from intensive care on the second day and hospital discharge 48 hours after the endovascular procedure with a hemoglobin of 9.2 g/dL and adequate functional recovery.

This case illustrates the complexity of an iatrogenic infrarenal abdominal aortic pseudoaneurysm that was initially contained by a retroperitoneal hematoma and remained subclinical for several months. The progressive deterioration of the patient and the final syncopal event led to the diagnosis by angiotomography, which showed the rupture of the pseudoaneurysm. The urgent endovascular approach, together with intensive hemodynamic management, effectively sealed the leak and stabilized the patient, highlighting the importance of a high index of clinical suspicion, the availability of advanced imaging techniques, and multidisciplinary collaboration for a successful outcome. With regard to the initially attempted laparoscopic cholecystectomy, the procedure was canceled intraoperatively due to hemodynamic instability and remains pending, with reprogramming planned once the patient achieves complete vascular recovery and clinical stability.

## Discussion

Currently, laparoscopic surgery is widely used in clinical practice due to its safety and efficacy; however, it is not without risk. A significant complication can involve a retroperitoneal vascular injury, which can occur in a small but variable percentage of procedures [6]. In hemodynamically stable patients with suspected traumatic aortic injury, computed tomography angiography (CTA) is the diagnostic tool of choice due to its rapid availability, high sensitivity, and ability to provide detailed anatomical information [7].

According to the Society for Vascular Surgery, aortic injuries can be classified into minor lesions, such as intimal disruption (grade I) and intramural hematoma (grade II), and more severe injuries, including pseudoaneurysm (grade III) and rupture (grade IV) [8,9]. The case presented here falls into the more severe category and requires urgent intervention.

Therapeutic options for such injuries include conservative management, endovascular or open repair. The indications for repair are also well-defined. The American College of Cardiology and American Heart Association guidelines recommend surgical repair of asymptomatic aneurysms of the ascending aorta and the aortic arch with a maximum diameter ≥5.5 cm. For descending thoracic aortic aneurysms (DTAAs), current guidelines recommend repair if the maximum diameter is ≥5.5 cm for endovascular repair or ≥6.0 cm for open surgical repair. Similarly, the European Society for Vascular Surgery advises intervention at 5.5 cm for patients who are suitable candidates for either endovascular or open repair. All patients with aneurysms >6.0 cm should be considered for repair if they are assessed to be at an acceptable operative risk. However, repair may not be feasible in those with excessive perioperative risk or unfavorable anatomical characteristics. Intervention is also indicated in cases of saccular thoracic aortic aneurysms, thoracic aortic pseudoaneurysms, and symptomatic aneurysms [7,8,10].

**Figure 4:**
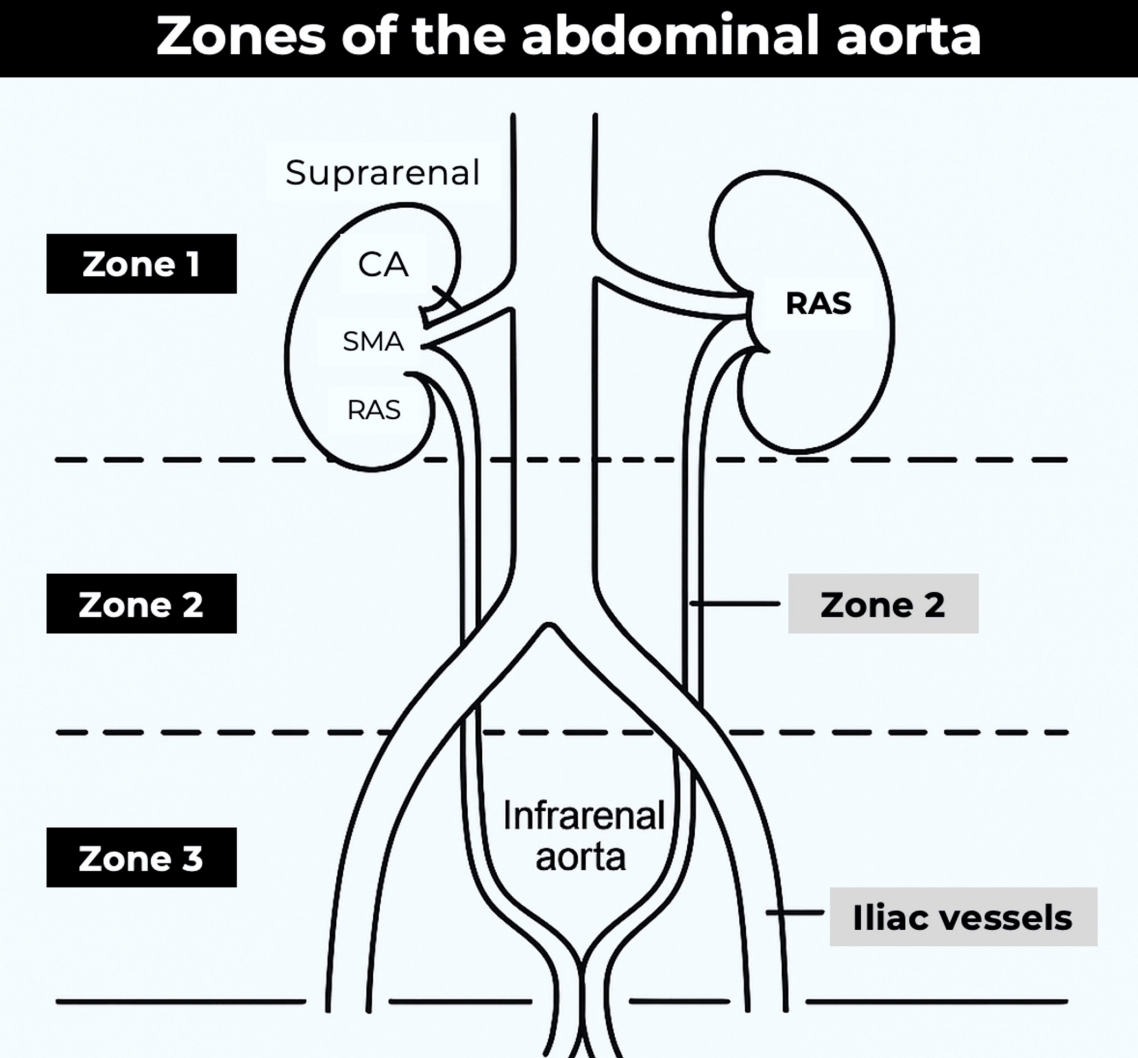
Schematic representation of the abdominal aortic zones used in vascular surgical planning. Zone I: midline retroperitoneum, extending from the aortic hiatus to the sacral promontory. This zone is subdivided into the supramesocolic (suprarenal aorta, CA, SMA, renal arteries, IVC, SMV) and inframesocolic areas (infrarenal aorta and IVC). Zone II: upper lateral retroperitoneum (left and right), containing the kidneys and their vessels. Zone III: pelvic retroperitoneum, including the iliac vessels. CA, celiac axis; SMA, superior mesenteric artery; RAS, renal artery stenosis Adapted from Karaolanis et al., 2018 [14]. Licensed under CC BY 4.0 (https://creativecommons.org/licenses/by/4.0/).

For surgical planning, vascular surgeons typically divide the abdominal aorta into three zones: Zone I extends from the diaphragmatic hiatus to the superior mesenteric artery (SMA); Zone II extends from the superior mesenteric to the renal arteries; and Zone III lies between the renal arteries and the aortic bifurcation [8,9]. Lesions in Zone III, as in the case described, can be treated with either open or endovascular reparative techniques. In particular, endovascular repair has demonstrated superior short- and mid-term outcomes compared to open surgery, underscoring its increasingly important role in contemporary vascular practice [11-13]. A schematic representation of these anatomical zones is presented (Figure 4) for visual reference. 

## Conclusions

Although rare, penetrating injuries to the abdominal aorta pose a significant clinical challenge due to their high risk of mortality. Early recognition and timely intervention are critical, as demonstrated in this case. Emergency endovascular management, particularly in scenarios such as aborted laparoscopic cholecystectomy due to intraoperative vessel injury, has proven to be a feasible and less invasive alternative to open repair, contributing to improved patient outcomes.

While endovascular repair offers significant short-term benefits, particularly in multi-trauma patients, it remains a technically challenging field as therapeutic strategies and device technologies continue to evolve. In addition, concerns remain about the long-term durability of endograft repairs. Recent multi-center studies by the American Association for the Surgery of Trauma (AAST), an organization that promotes trauma surgery and provides validated injury scoring systems, and the contributions by Demetriades et al. suggest a paradigm shift in the management of aortic injuries. These new findings highlight the need for further research to optimize treatment algorithms and confirm the long-term efficacy of endovascular interventions.

## References

[1] Pfabe FP (2020). The treatment of aneurysms of the extremities arteries - a systematic overview - new therapies for isolated iliac artery aneurysm employing a new classification (German). Zentralbl Chir.

[2] Peters S, Braun-Dullaeus R, Herold J (2018). Pseudoaneurysm. Hamostaseologie.

[3] Kuivaniemi H, Ryer EJ, Elmore JR, Tromp G (2015). Understanding the pathogenesis of abdominal aortic aneurysms. Expert Rev Cardiovasc Ther.

[4] Miller JB, Heitsch L, Madsen TE (2019). The extended treatment window’s impact on emergency systems of care for acute stroke. Acad Emerg Med.

[5] Aljarrah Q, Alomari M, Zureikat G (2021). Laparoscopic management of a rare cause of small bowel obstruction: a case report and literature review. J Curr Surg.

[6] Mihály Z, Öz T, Fernandez Prendes C, Stana J, Tilimparis N (2019). Diagnostik und Therapie des Bauchaortenaneurysmas in der Hausarztpraxis: Arterienerweiterungen im Blick haben. Dtsch Arztebl Int.

[7] Kurt D, Ammar C, Ablah E, Lightwine K, Okut H, Lu L, Haan JM (2023). Evaluation of outcomes and treatment options among trauma patients with abdominal vascular injuries. Kans J Med.

[8] Brown SR, Still SA, Eudailey KW, Beck AW, Gunn AJ (2021). Acute traumatic injury of the aorta: presentation, diagnosis, and treatment. Ann Transl Med.

[9] Shalhub S, Starnes BW, Tran NT (2012). Blunt abdominal aortic injury. J Vasc Surg.

[10] Shalhub S, Starnes BW, Brenner ML (2014). Blunt abdominal aortic injury: a Western Trauma Association multicenter study. J Trauma Acute Care Surg.

[11] Yei K, Mathlouthi A, Naazie I, Elsayed N, Clary B, Malas M (2022). Long-term outcomes associated with open vs endovascular abdominal aortic aneurysm repair in a Medicare-matched database. JAMA Netw Open.

[12] Tong MZ, Eagleton MJ, Roselli EE (2022). Outcomes of open versus endovascular repair of descending thoracic and thoracoabdominal aortic aneurysms. Ann Thorac Surg.

[13] Riambau V, Böckler D, Brunkwall J (2017). Editor’s choice - management of descending thoracic aorta diseases: clinical practice guidelines of the European Society for Vascular Surgery (ESVs). Eur J Vasc Endovasc Surg.

[14] Karaolanis G, Moris D, McCoy CC, Tsilimigras DI, Georgopoulos S, Bakoyiannis C (2018). Contemporary strategies in the management of civilian abdominal vascular trauma. Front Surg.

